# Novel synergistic antitumor effects of rapamycin with bortezomib on hepatocellular carcinoma cells and orthotopic tumor model

**DOI:** 10.1186/1471-2407-12-166

**Published:** 2012-05-04

**Authors:** Cun Wang, Dongmei Gao, Kun Guo, Xiaonan Kang, Kai Jiang, Chun Sun, Yan Li, Lu Sun, Hong Shu, Guangzhi Jin, Haiyan Sun, Weizhong Wu, Yinkun Liu

**Affiliations:** 1 Liver Cancer Institute, Zhongshan Hospital, Fudan University, Key Laboratory of Carcinogenesis and Cancer Invasion, Ministry of Education, Shanghai, 200032, China; 2 Institutes of Biomedical Sciences, Fudan University, Shanghai, 200032, China

**Keywords:** Rapamycin, Bortezomib, mTOR, Akt, Hepatocellular carcinoma

## Abstract

**Background:**

Despite recent advances in the treatment of hepatocellular carcinoma (HCC), the chemotherapy efficacy against HCC is still unsatisfactory. The mammalian target of rapamycin (mTOR) has been emerged as an important cancer therapeutic target. However, HCC cells often resistant to rapamycin because of the paradoxical activation of Akt by rapamycin. In this study, we investigated whether bortezomib could enhance the antitumor effects of rapamycin.

**Methods:**

The effects of rapamycin and bortezomib on HCC proliferation, apoptosis, migration, and invasiveness *in vitro* were assessed by CCK-8 analysis, flow cytometry, Hoechst 33342 staining and transwell assays, respectively. Total and phosphorylated protein levels of Akt were detected by Western blotting. The effects of rapamycin and/or bortezomib on the mRNA expression levels of p53, p27, p21 and Bcl-2 family in HCCLM3 cells were evaluated by RT-PCR. The roles of rapamycin and bortezomib on HCC growth and metastasis in xenograft models were evaluated by tumor volumes and fluorescent signals. The effects of rapamycin and bortezomib on cell proliferation and apoptosis *in vivo* were test by PCNA and TUNEL staining.

**Results:**

Bortezomib synergized with rapamycin to reduce cell growth, induce apoptosis, and inhibit cell mobility *in vitro*. Further mechanistic studies showed that bortezomib inhibited rapamycin-induced phosphorylated Akt, which in turn enhanced apoptosis of HCC cell lines. The alteration of the mRNA expression of cell cycle inhibitors p53, p27, p21 and apoptosis associated genes Bcl-2, Bax were also involved in the synergistic antitumor effects of rapamycin and bortezomib. P53 inhibitor PFT-α significantly attenuate the effect of rapamycin and bortezomib on cell apoptosis, which indicated that the pro-apoptotic effect of rapamycin and bortezomib may be p53-dependent. Treatment of HCCLM3-R bearing nude mice with rapamycin and bortezomib significantly enhanced tumor growth inhibition (72.4%), comparing with either rapamycin- (54.7%) or bortezomib-treated mice (22.4%). In addition, the lung metastasis was significantly suppressed in mice received the combination treatment (16.6%). The combination treatment of rapamycin and bortezomib significantly inhibited tumor cell proliferation and tumor angiogenesis *in vivo*.

**Conclusion:**

The combination of rapamycin with bortezomib could be a novel and promising therapeutic approach to the treatment of HCC.

## Background

Hepatocellular carcinoma (HCC) is the fifth most common solid tumor in the world and the third most common cause of cancer mortality [1]. To date, surgical resection and liver transplantation surgery is still the only potential curative treatment for patients with early-stage tumors (Barcelona Clinic Liver Cancer classification, BCLC 0 and A). However a considerable number of patients with advanced HCC are not suitable for surgery because of metastases [2]. Systemic pharmacotherapy is the main treatment for those patients (BCLC stage C and D). Unfortunately, traditional chemotherapy treatments show little efficacy in patients with advanced HCC and often have no survival benefit [3,4].

Recently, drugs that targeting key pathways have generated new perspectives in the field of the treatment of HCC. Sorafenib is the first and only systemic therapy to significantly prolong the survival of HCC patients with advanced-stage disease [5]. However, the tumor response rates to the treatment of are usually low [6]. And emerging preclinical findings from our institute revealed that invasiveness and metastatic behavior of tumor cells are increased after a long period treatment of sorafenib. Therefore, novel and effective pharmacological strategies for the treatment of advanced HCC are critically needed.

The mTOR pathway is aberrant activated in a proportion of HCC [7]. Inhibition of mTOR had been shown to suppress the liver tumor growth and metastasis [8,9]. Emerging clinical evidence as well as preclinical findings revealed that the mTOR inhibitor rapamycin and its analogues can be used in treating solid tumors, such as esophageal squamous cell carcinoma [10], lung cancer [11], renal cell carcinoma [12] and prostate cancer [13]. These reports imply that mTOR pathway could be a promising target for the treatment of HCC. Although rapamycin has shown preclinical promise, it exhibited little activity in HCC patients and the clinical outcomes are mostly unpredictable when rapamycin used as single agent. Recently, more studies have found that rapamycin could paradoxically activate Akt while blocking mTOR, which is a crucial mechanism accounting for rapamycin resistance. Sun *et al.* demonstrated that treatment of human lung cancer cells with rapamycin concurrently increased the phosphorylation of both Akt and eIF4E [14]. It also has been reported that mTOR inhibition will enhance insulin receptor substrate-1 expression and abrogate feedback inhibition of the pathway, resulting in Akt activation both in cancer cell lines and in patient tumors [15]. Moreover, disrupting mTORC1 by rapamycin may induce mTORC2 activation which is important for Akt phosphorylation [16]. The activation of Akt survival pathway can promote cell survival and inhibit apoptosis by a variety of routes [17]. Therefore we hypothesized that the combined use of an agent which can prevent Akt activation may potentialize the antitumor activity of rapamycin.

Bortezomib is the first clinically available proteasome inhibitor, which is often used in the treatment of hematological malignancies [18]. Multiple clinical trials have demonstrated that this small molecule possesses antitumor activity in a variety of human cancers, including HCC [19,20]. A multicenter, single-arm, phase II trial that evaluates the activity of bortezomib in HCC has been already conducted [21]. It is well known that bortezomib can exert its antitumor activity against cancer cells through inhibition of NF-КB activation by preventing IКB degradation [22]. Accumulating studies indicate that down-regulation of p-Akt is another potential mechanism of bortezomib-induced apoptosis in HCC cells [19]. Bortezomib down-regulates p-Akt in a dose- and time-dependent manner, which may be mediated by protein phosphatase 2A (PP2A) and cancerous inhibitor of protein phosphatase 2A (CIP2A) [23,24]. A combination therapy of bortezomib with sorafinib or tumor necrosis factor significantly down-regulates the expression of p-Akt and induces apoptosis of HCC cell lines [24,25]. Previous study has shown that mTOR inhibitors could have a role in combination with weekly bortezomib for the treatment of patients with relapsed and refractory multiple myeloma [26]. However there are no available clinical data on the combination of bortezomib and mTOR inhibitors on solid tumors.

In this study, we investigated the efficacy of the combination of rapamycin and bortezomib in HCC cells and orthotopic tumor model with the aim of developing novel HCC treatment approach.

## Methods

### Cell lines and materials

HCCLM3, a human HCC cell line with high metastatic potential that originated from MHCC97, was established by the Liver Cancer Institute of Fudan University (Shanghai, China) [27]. Stable red fluorescent protein-expressing HCCLM3 (HCCLM3-R) cells by infection with lentivirus containing full-length cDNA of red fluorescent protein were also established by our institute [28]. SMMC7721 was established by the Shanghai Institute of Cell Biology, Chinese Academy of Sciences. The cells were maintained at 37°C with a 5% CO_2_ in DMEM supplemented with 10% fetal bovine serum and antibiotics (100 U/ml penicillin, 100 mg/ml streptomycin). Rapamycin and bortezomib were purchased from LC Lab (Woburn, MA). Both drugs were dissolved in DMSO, and the final concentration of DMSO in the cell culture studies was 0.1% or less. Most of the *in vitro* assays were performed use the following concentration: rapamycin (10 ng/ml) and Bortezomib (100 nM) or indicated otherwise. The concentrations of rapamycin and bortezomib were based on previous study [8,24]. Chemical inhibitor of p53, pifithrin-α (PFT-α) was purchased from Santa Cruz Biotechnology (Santa Cruz, CA). Antibodies for western blot such as anti-Akt, anti-p-Akt Ser473 and anti-GAPDH were purchased from Cell Signaling Technology (Danvers, MA). Other antibodies such as anti-PCNA, anti-CD31 were obtained from Abcam (Hong Kong, China). Cell Counting kit and colorimetric TUNEL system were purchased from Dojindo (Kumamoto, Japan) and Promega (Madison, WI), respectively.

### Cell proliferation assay

To determine the effect of rapamycin and bortezomib on cell proliferation, we used a tetrazolium reagent, 2-(4-indophenyl)-3-(4-nitrophenyl)-5-(2,4-disulphophenyl)-2 H-tetrazolium monosodium salt (CCK8, Cell Counting kit). In brief, 1 **×** 10^3^ cells were seeded in 96-well culture plates. After an attachment period of 24 h, the cells were cultured in the presence of vehicle, rapamycin (10 ng/ml), bortezomib (100 nM), or a combination of both for 72 h. At the time of 24 h, 48 h and 72 h, the cells were incubated with CCK8 reagent for 1 h at 37°C. The staining intensity in the medium was measured by determining the absorbance at 450 nm.

### Cell cycle analysis

HCCLM3 and SMMC7721 cells plated on 20-cm^2^ tissue culture flasks were collected at 24 h after the incubation with vehicle, rapamycin (10 ng/ml), bortezomib (100 nM), or the combination of rapamycin and bortezomib. Then, the cells were fixed in 70% cold ethanol for 1 h and resuspended in a hypotonic propidium iodide (PI) solution (Sigma, St Louis, MO) containing RNase. Flow cytometry was performed with the use of Coulter epic flow cytometer. DNA histograms were analyzed using Modfit computer program (Verity Software House, Topsham, ME, USA). In addition, the percentage of apoptotic cells in the sub-G1 fraction was also calculated.

### Analysis of apoptotic cells by Hoechst 33342 staining

The chromatin dye Hoechst 33342 was used to assess apoptotic cells. The cells were incubated with vehicle, rapamycin (10 ng/ml), bortezomib (100 nM), or the combination of rapamycin and bortezomib for 24 or 48 h. After washing three times with PBS, the cells were fixed in 4% formaldehyde at room temperature for 30 min, then washed twice with PBS and exposed to 5 μg/ml Hoechst 33342 for 30 min at room temperature. Afterwards, the cells were observed with a fluorescence microscope. Apoptotic cells were characterized by morphological alterations such as condensed nuclei and cell shrinkage.

### Cell migration assay and cell invasion assay

Cell migration was performed by transwell assay (Corning Costar, Cambridge, MA). The cells were incubated with vehicle, rapamycin (10 ng/ml), bortezomib (100 nM), or the combination of rapamycin and bortezomib for 24 h. Then 5 × 10^4^ pretreated cells in serum-free DMEM were seeded on a membrane (8.0-μm pore size) inserted in a well of a 24-well plate. DMEM containing 10% FBS was added to the lower chamber of each well. After 48 h, cells in the upper chamber were removed by cotton swab and the cells that had reached the underside of the membrane were fixed with 4% paraformaldehyde and stained with 10% Giemas for 10 min. The cells that located on the underside of the filter (5 fields/filter) were counted. The cell invasion assay was carried out similarly, except that the matrigel (BD Biosciences) was added to each well 6 h before cells were seeded on the membrane.

### Western blot analysis

Equal amounts of total proteins (20 μg) were separated by 10% SDS-PAGE and transferred to a 0.45-mm PVDF membrane using a Bio-Rad SemiDry apparatus. The membranes were blocked at room temperature for 1 h with 5% nonfat milk in TBS containing Tween 20 (TBST). Then membranes were incubated with rabbit anti-Akt, rabbit anti-p-Akt Ser473, or mouse anti-GAPDH overnight at 4°C, followed by HRP-conjugated secondary antibodies for 1 h at room temperature. After washing three times in TBST, the membrane was incubated with Chemiluminescent Detection Reagent for 5 min and exposed to X-ray film.

### RT-PCR

Total RNA was extracted using Trizol Reagent (Invitrogen, Carlsbad, CA) and was reverse-transcribed into complementary DNA with oligo (dT)18 primer using TransScript first-strand cDNA synthesis kit (Transgene Biotech, China) according to the manufacturer’s instructions. PCR was performed with PCR kit (Lifefeng, China) according to the manufacturer’s instructions. The primer sequences, annealing temperature, number of cycles used for PCR, and length of the amplified products can be seen in Additional file 1: Table S1. The PCR products were analyzed on 1.5% agarose gel and visualized under UV light following ethidium bromide staining. Quantitative data were expressed by normalizing the densitometric units to GAPDH (internal control).

### Orthotopic tumor model

Male BALB/C nude mice (5- to 6-week-old) were obtained from Shanghai Institute of Materia Medica (Chinese Academy of Sciences, Shanghai, China). The *in vivo* experiments were carried out strictly in accordance with a protocol approved by the Shanghai Medical Experimental Animal Care Committee (Permit Number: 2009–0082). HCCLM3-R hepatocellular cancer orthotopic xenograft model was established as described previously [29,30]. Briefly, HCCLM3-R cells (5 × 10^6^) were injected subcutaneously into the upper left flank region of nude mice. When the tumor reached 1 cm in diameter, they were cut into 2 × 2 × 2 mm^3^ sized pieces, and implanted into livers of 24 nude mice. The 24 mice were randomly assigned to four experimental groups (6 mice/group): control, rapamycin, bortezomib, and the combination of rapamycin with bortezomib. Both drugs were dissolved in DMSO and then diluted in saline, and the final concentration of DMSO in the *in vivo* studies was less than 0.1%. On day 4 after the tumor implantation, rapamycin was delivered orally at a dose of 2 mg/kg every day. Bortezomib was administered as i.p. injections at a dose of 0.5 mg/kg in 100 μL of 0.9% saline solution twice a week. The dosage of rapamycin and bortezomib is based on previous studies [9,25]. The mice treated with both rapamycin and bortezomib was administered by using the same schedule as described for the single drug treatment. Control mice were received 100 μL of saline orally (every day) and 100 μL of saline by i.p. injections (twice a week) including the same concentration of DMSO with the treatment groups.

The treatment was stopped on day 33. Then the mice were sacrificed on day 35 after tumor implantation. At necropsy, tumor was measured for largest (a) and smallest (b) diameters, and the tumor volume was calculated as V = a × b^2^/2. The lungs were excised, and red fluorescent protein-positive metastatic foci were imaged by fluorescent imaging (Leica MZ6). Then, orthotopic tumors and lungs were fixed with 10% buffered formalin and embedded with paraffin. Lung metastasis was also determined by examining serial sections of every lung tissue block by microscopy.

### Immunohistochemistrical staining for Akt, p-Akt, PCNA and CD31

Tumor tissue was fixed, embedded, and sliced into 5 μm thick sections. Immunostaining of Akt, p-Akt Ser473, PCNA and CD31 was carried out as described previously [31]. Briefly, paraffin sections were first deparaffinized and then hydrated. After microwave antigen retrieval, endogenous peroxidase activity was blocked with incubation of the slides in 0.3% H_2_O_2_, and non-specific binding sites were blocked with 10% goat serum. Then the sections were incubated with primary antibodies (rabbit anti-Akt, rabbit anti-p-Akt Ser473, rabbit anti-PCNA and mouse anti-CD31) overnight at 4°C, followed by HRP-conjugated secondary antibodies for 1 h at room temperature. Finally, the sections were developed in diaminobenzidine solution under a microscope and counterstained with hematoxylin. The proliferation index was determined as number of PCNA-positive cells/total number of cells in 5 randomly selected high-power fields (magnification × 200).

### *In situ* apoptosis detection by TUNEL staining

Paraffin-embedded, 5 μm thick sections were used to identify apoptotic cells by staining using TUNEL assay kit according to the manufacturer’s instructions. The extent of apoptosis was evaluated by counting the TUNEL-positive cells (brown-stained). The apoptotic index was determined as number of TUNEL-positive cells/total number of cells in 5 randomly selected high-power fields (magnification × 200).

### Statistics

Analysis was performed with SPSS 15.0 for Windows. *In vitro* cell proliferation, apoptosis migration and invasion assays were analyzed by Student's *t*-test. Tumor volumes and histologic quantitations were also compared by Student’s *t* test. Categorical variables were tested with the use of Fisher’s exact test. *P* <0.05 were considered statistically significant.

## Results

### Combined treatment with rapamycin and bortezomib inhibits HCC cell proliferation

**Figure 1:**
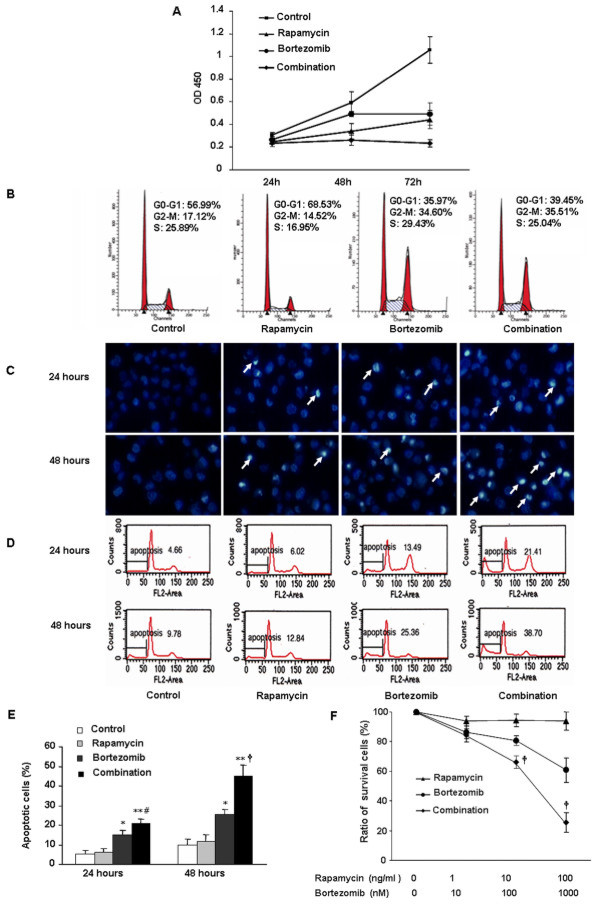
** Combined treatment with rapamycin and bortezomib inhibited cell proliferation and enhanced cell apoptosis.** (**A**) Proliferation of HCCLM3 cells was evaluated by using Cell Counting Kit-8 (CCK-8) at indicated time points. (**B**) HCCLM3 cells were treated with rapamycin (10 ng/ml), bortezomib (100 nM) or both agents. ModFit software analysis of flow cytometry histograms revealed that rapamycin treatment resulted in cell cycle arrest at G1-S phase. Bortezomib significantly increased in the percentage of cells in the G2/M phase. When the two agents were combined, no significant difference in cell cycle distribution was observed compared with bortezomib alone. (**C**) HCCLM3 cells were treated with rapamycin (10 ng/ml), bortezomib (100 nM) or the combination for 24 or 48 h and stained with Hoechst 33342, the apoptotic nuclear changes (arrows) were examined by fluorescence microscopy (magnification, ×400). (**D**, **E**) The quantification of apoptotic cells induced by rapamycin and bortezomib was further confirmed by flow cytometry analysis. The sub-G1 contents were designed as apoptotic cells. ^*^P < 0.01, *versus* control group; ^**^P < 0.001, *versus* control group; ^#^*P* < 0.05, *versus* bortezomib treatment group at 24 h; ^†^*P* < 0.01, *versus* bortezomib treatment group at 48 h. (**F**) The cells were treated with various concentrations of the drugs for 24 h, and then evaluated by using Cell Counting Kit-8. ^†^*P* < 0.01, *versus* bortezomib treatment group.

CCK8 analysis was performed to test the effect of rapamycin and bortezomib on proliferation of HCCLM3 and SMMC7721 cells. The cells were cultured in the presence of vehicle, rapamycin (10 ng/ml), bortezomib (100 nM), or a combination of rapamycin and bortezomib for 24, 48, and 72 h, respectively. Growth of HCCLM3 and SMMC7721 was significantly inhibited by each drug alone. The combination of both drugs further decreased the proliferation rate of HCCLM3 and SMMC7721, compared to single drug application. The inhibition rates of HCCLM3 and SMMC7721 in the combined treatment group at 72 h were 77.9% and 69.3%, respectively (Figure 1A and Additional file 2: Figure 1A).

Then, we examined whether treatment of cells with rapamycin and/or bortezomib would change cell cycle by single-cell analysis using flow cytometry. Rapamycin (10 ng/ml) did block G1 to S transition of HCCLM3 and SMMC7721 cells. Bortezomib (100 nM) induced cell cycle arrest at the G2-M phase. Similar results were observed upon treatment of the cells with rapamycin plus bortezomib (Figure 1B, Additional file 2: Figure S1B).

### Combined treatment with rapamycin and bortezomib induces cell apoptosis

HCCLM3 cells were cultured in the presence or absence of rapamycin, bortezomib, or their combination for 24 and 48 h, respectively. Apoptosis of those cells was assessed with Hoechst 33342 staining and FACS analysis. As shown in Figure 1C, most cells from the control had big, regular nuclei, with only a few cells showing apoptotic nuclei with condensed chromatin by Hoechst 33342 staining analysis. Rapamycin did not induce apoptosis of HCCLM3 cells, but bortezomib did. When the cells were cultured in the presence of both rapamycin and bortezomib, rapamycin potentiated the apoptotic effect of bortezomib from 16.4 ± 5.8% to 27.4 ± 6.2% (*P* < 0.05) at 24 h and 30.7 ± 7.4% to 53.6 ± 9.4% (*P* < 0.01) at 48 h.

To further confirm the effect of rapamycin and bortezomib on cell apoptosis, FACS analysis was used to assess apoptosis by detection of sub-G1 phase through PI staining. As shown in Figure 1D, rapamycin did not significantly induce apoptosis of HCCLM3 cells at 24 or 48 h compared with the control (*P* > 0.05). However, bortezomib significantly induced 15.2 ± 2.1% of HCCLM3 cells to apoptotic death at 24 h and 25.5 ± 2.3% at 48 h. In comparison with bortezomib alone, the combination of rapamycin and bortezomib significantly enhanced apoptosis of HCCLM3 cells to 20.7 ± 2.4% at 24 h and 44.8 ± 5.8% at 48 h (Figure 1D and E).

Then different concentrations of single agent or in parallel were used to further confirm the synergistic activity of the combined treatment through CCK8 assay. Rapamycin, as single agent, did not induce apoptosis of HCCLM3 cells. Bortezomib induced cell apoptosis in dose dependent fashion. Combination of rapamycin and bortezomib caused synergistic effects on cell apoptosis (Figure 1F). In comparison with bortezomib alone, the synergistic effects of rapamycin and bortezomib was noted to be significantly different from single agent treatment at bortezomib concentrations of 100 and 1000 nM (*P* < 0.01).

Then we used another HCC cell line SMMC 7721 to further validate the synergistic effect of rapamycin and bortezomib. As shown in Additional file 3: Figure S2, rapamycin did not induce significant apoptosis of SMMC7721 cells at 24 or 48 h. Treatment of SMMC7721 cells with rapamycin and bortezomib significantly increased bortezomib induced cell apoptosis at 48 h, even though bortezomib alone have significantly increased apoptotic cell death.

### Effects of rapamycin and bortezomib on migration and invasion of HCCLM3

**Figure 2:**
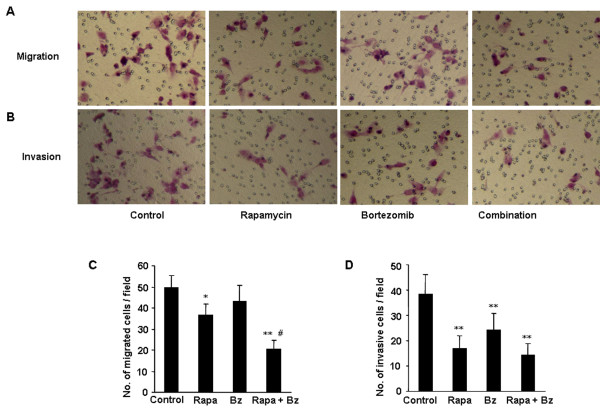
** Effects of rapamycin and bortezomib on migration and invasion of HCCLM3.** (**A**, **C**) The transwell assay demonstrated that rapamycin and combined treated cells migrated through the membrane in less number compared with control group. The inhibitory effect of migration was markedly enhanced by combined treatment. (**B**,** D**) Rapamycin or bortezomib significantly inhibited cell invasion, as compared with control group. No significant difference was observed between rapamycin treatment alone and combined treatment (magnification, ×400). ^*^P < 0.05, *versus* control group; ^**^P < 0.01, *versus* control group; ^#^*P* < 0.01, *versus* rapamycin treatment group.

Migration and invasion assay *in vitro* were carried out to evaluate the effects of rapamycin and bortezomib on HCCLM3 cell mobility. In the migration assay, 10 ng/ml of rapamycin significantly inhibited migration of HCCLM3 cells compared with the control (*P* < 0.05, Figure 2A and C). In contrast, bortezomib alone, at 100 nM, did not significantly affect on HCCLM3 cell migration. The suppression of transwell migration was significantly greater in the combination of rapamycin and bortezomib in comparison with rapamycin alone (*P* < 0.01, Figure 2A and C). In the invasion assay, rapamycin or bortezomib significantly reduced the number of pretreated HCCLM3 cells invading the matrigel coated membrane, with 2.29-fold and 1.58-fold decrease, respectively, compared with the untreated controls (*P* < 0.01, Figure 2B and D). The combination of rapamycin and bortezomib didn’t affect cell invasion further compared with either single agent (Figure 2B and D).

### Rapamycin induces p-Akt in HCCLM3 cells, whereas bortezomib suppresses p-Akt

**Figure 3:**
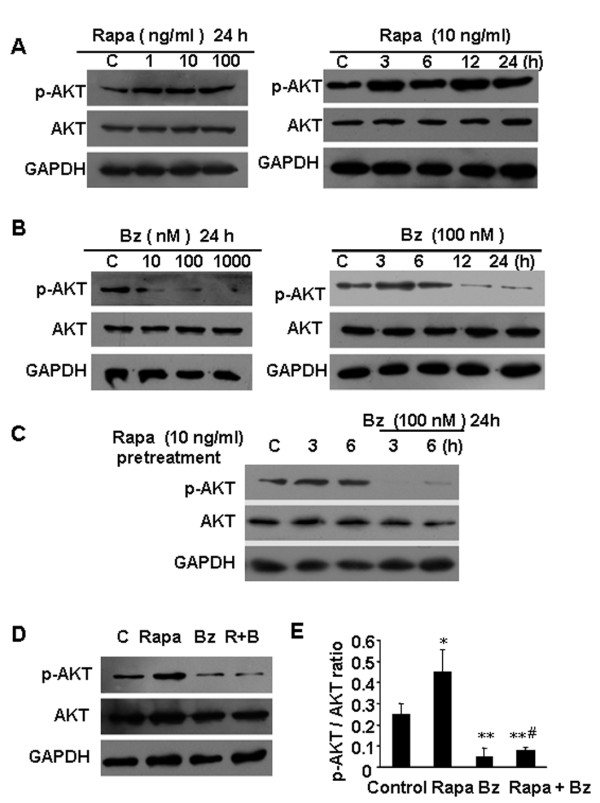
** Rapamycin induced p-Akt in HCCLM3 cells, whereas bortezomib inhibited p-Akt.** (**A**) HCCLM3 cells were incubated with culture media or rapamycin (1–100 ng/ml) for 24 h or rapamycin (10 ng/ml) for the indicated periods. (**B**) HCCLM3 cells were incubated for 24 h with culture media, bortezomib (10–1000 nM), or bortezomib (100 nM) for the indicated time. (**C**) Rapamycin is added to HCC cells for 3–6 h prior to the addition of bortezomib, and then the cells were cultured for 24 h in the presence of bortezomib at the concentration of 100 nM. (**D**, **E**) HCCLM3 cells were cultured in control media, rapamycin (10 ng/ml), bortezomib (100 nmol/L), or the combination of rapamycin and bortezomib for 24 h. ^*^P < 0.05, *versus* control group; ^**^P < 0.01, *versus* control group; ^#^*P* < 0.001, *versus* rapamycin treatment group.

To confirm the effects of rapamycin on the expression of p-Akt in HCCLM3 cells, the cells were exposed to increasing concentrations of rapamycin for 24 h. Rapamycin increased phosphorylation of Akt at Ser473, starting at doses as low as 1 ng/ml. Activation of p-Akt was observed as early as 3 h after exposure of HCCLM3 cells to 10 ng/ml of rapamycin and this effect can last at least for 24 h (Figure 3A). To examine the effects of bortezomib on p-Akt in HCCLM3 cells, the cells were cultured in the presence of increasing doses of bortezomib (10–1000 nM) for 24 h. As shown in Figure 3B, bortezomib suppressed p-Akt in a dose-dependent manner. Although a slight activation of p-Akt was observed at 3 h exposure of HCCLM3 cells to 100 nM of bortezomib, bortezomib suppressed p-Akt when the exposed time prolonged.

The p-Akt level was tested when rapamycin was added to HCC cells for 3–6 h prior to the addition of bortezomib. We found that rapamycin-mediated Akt phosphorylation could be significantly suppressed after the cells were cultured for 24 h in the presence of bortezomib at the concentration of 100 nM (Figure 3C). Next, we incubated the cells with rapamycin (10 ng/ml), bortezomib (100 nM), or the combination of both agents for 24 h to study their effects on p-Akt. Rapamycin increased p-Akt, which was abrogated by bortezomib in the combined treatment. There was no significant change in total Akt expression under those treatment conditions (Figure 3D and E). Similar results were also observed in SMMC7721 cells (Additional file 4 Figure S3).

### The pro-apoptotic effect of rapamycin plus bortezomib is dependent on p53 activity

**Figure 4:**
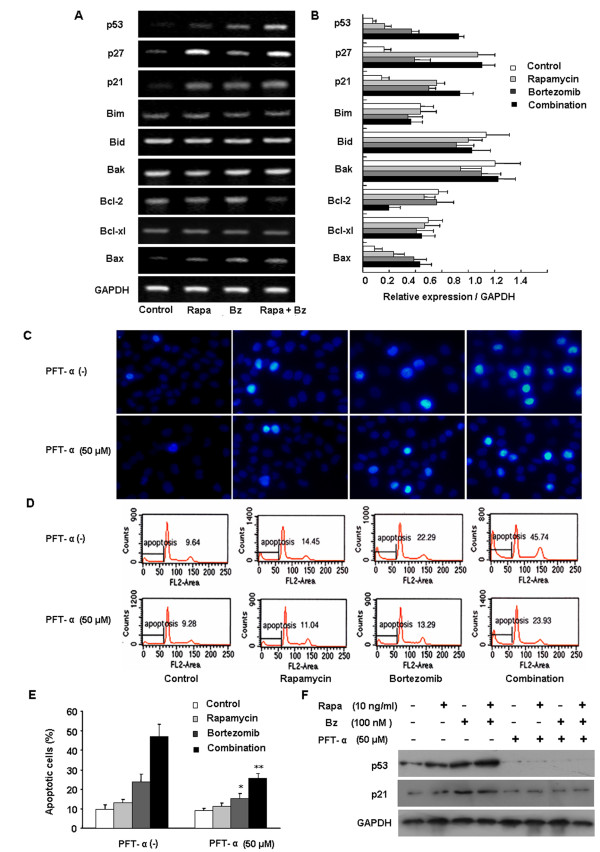
** The pro-apoptotic effect of rapamycin plus bortezomib is dependent on p53 activity.** (**A, B**) The mRNA expression levels of p53, p27, p21 and Bcl-2 family in HCCLM3 cells were evaluated by RT-PCR. Bcl-2 mRNA was significantly suppressed by the combined treatment of rapamycin and bortezomib. The expression of p53, p27 p21and Bax mRNA was significantly up-regulated by monotherapy or the combination therapy. (**C**) HCCLM3 cells were treated with rapamycin (10 ng/ml), bortezomib (100 nM) or the combination with or without PFT-α for 48 h and stained with Hoechst 33342, the apoptotic nuclear changes were examined by fluorescence microscopy (magnification, ×400). (**D**, **E**) The quantification of apoptotic cells was further confirmed by flow cytometry analysis. The sub-G1 contents were designed as apoptotic cells. (**F**) The rapamycin plus bortezomib mediated up-regulation of p53 protein could be significantly suppressed by p53 inhibitor PFT-α. And the level of p21 protein was also down-regulated during this process. ^*^P < 0.05, *versus* bortezomib group without PFT-α treatment; ^**^P < 0.01, *versus* combined treatment group without PFT-α.

The effects of rapamycin and/or bortezomib on the mRNA expression levels of p53, p27, p21 and Bcl-2 family in HCCLM3 cells were evaluated by RT-PCR. As shown in Figure 4, the level of p53, p27, p21 or Bax mRNA expression was significantly up-regulated after the treatment of HCCLM3 cells with either single agent or the combination of both agents as compared with the control. The mRNA level of Bcl-2 was significantly suppressed by the combination treatment. Rapamycin and/or bortezomib did not affect the expression levels of other Bcl-2 family members, Bim, Bid, Bak, and Bcl-xl (Figure 4A and B).

Following the discovery that rapamycin plus bortezomib increased p53 mRNA expression in HCCLM3 cells, we determined whether the pro-apoptotic effect was dependent on p53 activity. HCCLM3 cells treated with the p53 inhibitor PFT-α abrogated p53 expression induced by either single agent or the combination of both agents. And the level of p21 protein was also down-regulated during this process (Figure 4F). As shown in Figure 4D and E, HCCLM3 cells were treated with rapamycin and/or bortezomib together with PFT-α for 48 h, the number of apoptotic cells was markedly decreased in the presence of PFT-α from 23.8 ± 3.7% to 15.1 ± 2.5% in bortezomib treatment group (*P* < 0.05) and 47.0 ± 6.4% to 25.5 ± 2.6% in the combined treatment group (*P* < 0.01). The number of apoptotic cells did not obviously change in rapamycin group during PFT-α treatment. Similar results were also observed from Hoechst 33342 staining.

### *In vivo* effects of rapamycin, bortezomib, or the combination on tumor growth and lung metastasis

**Figure 5:**
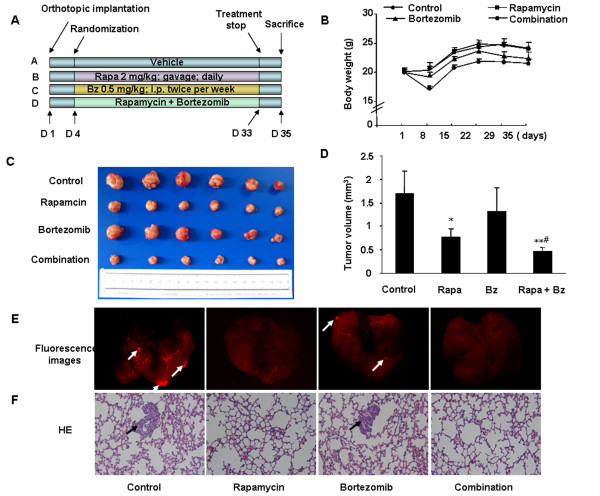
** The synergistic antitumor effects of rapamycin with bortezomib on orthotopic tumor model.** (**A**) Schematic representation of the experimental protocol as described in materials and methods. (**B**) Body weight was recorded to assess toxicity of treatment. (**C**) Tumors of mice from each group on 5 wk after tumor implantation. (**D**) Average tumor volume of each group on 5 wk after tumor implantation. ^*^P < 0.01, *versus* control group; ^**^P < 0.001, *versus* control group; ^#^*P* < 0.05, *versus* rapamycin treatment group. (**E**) The representative fluorescence images of pulmonary metastasis (arrows) from each group on 5 wk in HCCLM3-R xenograft model (magnification, ×10). (**F**) The representative HE staining of pulmonary metastasis (arrows) on 5 wk in HCCLM3-R xenograft model (magnification, ×200).

In order to determine whether the synergistic effect of rapamycin and bortezomib in HCCLM3 cell line has relevant clinical implications, we used an orthotopic tumor model to test the *in vivo* effect of rapamycin and/or bortezomib on the tumor growth and lung metastasis of HCC xenograft tumors. Rapamycin significantly inhibited tumor growth with average tumor volume of 0.77 ± 0.18 cm^3^, in comparison with 1.70 ± 0.57 cm^3^ of the control mice (*P* <0.01). Bortezomib decreased the tumor volume to 1.32 ± 0.54 cm^3^, but did not significantly differ with the control (Figure 5C and D). The combination of rapamycin and bortezomib significantly improved the tumor inhibitory effect of rapamycin, with average tumor volume of 0.47 ± 0.07 cm^3^ (*P* < 0.05, Figure 5C and D). The rate of tumor growth inhibition index by the combination treatment was 72.4%, significantly higher than the rate in the single agent group.

Toxicity observed with the combination treatment of rapamycin and bortezomib was evidenced by 20% weight loss at day 5 after initiation of treatment, which was reversed after completion of treatment (Figure 5B). And no significant abnormalities were found in these mice as evaluated pathologic examinations of heart, liver, and kidney tissues.

The presence of lung metastasis was determined by both fluorescent imaging and HE staining. While 100% of mice in the control group developed pulmonary metastasis, rapamycin or bortezomib treatment caused a reduction of the pulmonary metastasis of 50% and 33.4%, respectively (Figure 5E and F). However, the difference of pulmonary metastasis percentage between the control group and either single agent group did not reach statistical significance (*P* > 0.05). The combination treatment of rapamycin and bortezomib significantly suppressed pulmonary metastasis, with only one out of six mice (16.6%) developed pulmonary metastasis (*P* < 0.05, Fisher’s Exact test *versus* control group). Then we counted the lung metastasis nodules of each mouse. We found that the median number of pulmonary nodules in rapamycin and combined treatment group is 17.5 and 0, respectively, which is obvious suppressed compared with control group (Additional file 5: Table S2).

### Effects of rapamycin and bortezomib alone or in combination on proliferation, angiogenesis, and apoptosis in HCC

**Figure 6:**
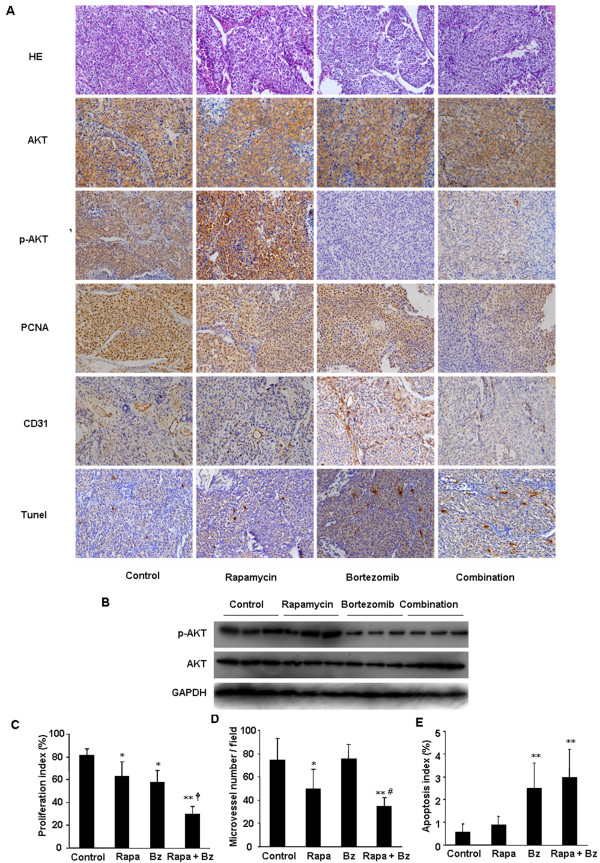
*** In vivo* mechanism of antitumor activity of rapamycin and bortezomib in xenograft model.(A)***Ex vivo* immunohistochemistry staining of Akt, p-Akt, PCNA, CD31 or TUNEL in HCCLM3-R xenografts (magnification, ×200). The staining of p-Akt was increased in rapamycin-treated tumors, but inhibited in bortezomib- or the combination-treated tumors. **(B)** The increase of p-Akt in rapamycin-treated tumors and the decrease in bortezomib-treated tumors were also observed by Western blot analysis. Rapamycin-induced Akt phosphorylation was abrogated by bortezomib in the combination-treated tumors. **(A, C)** Quantitation of PCNA positive cells demonstrated that rapamycin or bortezomib significantly inhibited tumor growth, as compared with control (^*^P < 0.05), which was markedly enhanced by the combination of both agents (^**^P < 0.01, *versus* control group and ^†^*P* < 0.01 *versus* monotherapy). **(A, D)** The average number of CD31-positive vessels was significantly reduced in tumors from the rapamycin monotherapy group (^*^P < 0.05, *versus* control group), which was further enhanced by the combined therapy of rapamycin and bortezomib (^**^P < 0.01, *versus* control group; and ^#^*P* < 0.05, *versus* rapamycin group). **(A, E)** The number of TUNEL-positive cells in tumors was significantly elevated in bortezomib treatment group (^**^P < 0.01, *versus* control group) and the combined treatment group (^**^P < 0.01, *versus* control group or rapamycin group).

To confirm the mechanism identified *in vitro*, the effect of rapamycin and bortezomib on Akt phosphorylation *in vivo* was assayed by immunohistochemistry and western blotting. Similar to the *in vitro* experiments, rapamycin increased the level of p-Akt in HCC tumors after 30 days of treatment, which was abrogated by bortezomib in the combination therapy (Figure 6A). Three HCCLM3-R tumors from each treatment group were further examined for Akt phosphorylation by western blotting. As shown in Figure 6B, p-Akt was increased in the HCCLM3-R tumors treated with rapamycin, but decreased with bortezomib treatment. The combination of rapamycin and bortezomib inhibited p-Akt in HCCLM3-R tumors (Figure 6B).

To investigate whether rapamycin alone or in combination with bortezomib decrease HCC tumor growth by inhibiting cell proliferation, we examined the number of PCNA-positive cells in HCCLM3-R tumors from mice with different treatments. The proliferation index in the control group was 81.2 ± 5.8%. Rapamycin or bortezomib significantly decreased tumor cell proliferation index to 62.8 ± 12.7% and 57.8 ± 10.6%, respectively (*P* < 0.05, *versus* the control group). The combination treatment of rapamycin and bortezomib was significantly more effective in reducing PCNA expression (29.8 ± 6.5%) than either agent alone (*P* < 0.01, *versus* monotherapy group, Figure 6A and C).

The expression of the microvessel density marker CD31 in orthotopic tumors from nude mice in different treatment groups was also examined. The average number of CD31-positive vessels in the control group was 74.5 ± 18.5. Rapamycin reduced HCC vascularization with 49.5 ± 16.9 of CD31-positive vessel in tumors (Figure 6A and D). Although bortezomib alone did not affect tumor vascularization, the combination of bortezomib with rapamycin significantly decreased the number of CD31-positive vessels to 35 ± 7.0 (*P* < 0.01, *versus* the control; *P* < 0.05, *versus* rapamycin alone) (Figure 6A and D).

Furthermore, the effect of rapamycin and/or bortezomib on tumor cell apoptosis was examined by TUNEL. The average number of apoptosis index was 0.56 ± 0.37% in the control tumors. Bortezomib significantly increased the number of apoptosis index to 2.52 ± 1.10%, compared with the control mice (*P* < 0.01, Figure 6A and E). The apoptosis index was not significant different between the rapamycin-treated group (0.88 ± 0.38%) and the control group. The combination therapy of rapamycin and bortezomib increased apoptosis index to 2.97 ± 1.24%, higher than that in bortezomib alone, but without statistical significance (Figure 6A and E).

## Discussion

Due to unsatisfactory outcomes of available chemotherapies, new therapeutic strategies for HCC are urgently needed. More effective treatments may involve combination of agents with synergistic activity against HCC. Rapamycin, a mTOR inhibitor, has been used in treating various solid tumors. Despite its outstanding preclinical antitumor activity, rapamycin has been shown to increase Akt phosphorylation, which may counteract its anticancer efficacy [14,15] And could be a reason why mTOR inhibitors only exhibit modest antitumor activity in patients. In this study, we hypothesized that the combined use of an agent which down-regulates Akt survival pathway would potentiate the antitumor effects of rapamycin in HCC cells. Focus on the activation of Akt caused by mTOR inhibition, there are some novel therapeutic approaches [32-34]. In our study, we chose the combination of rapamycin with bortezomib for a couple of reasons. Firstly, rapamycin and bortezomib have shown single-agent activity in preclinical studies in HCC. Secondly, PI3K/Akt/mTOR pathway is critical for cell survival and resistance to apoptosis and both agents interact at the PI3K/Akt/mTOR pathway [10,19], which is known to be activated in HCC. Our results in this study have demonstrated that suppression of mTOR signaling by rapamycin in HCCLM3 cells was associated with up-regulation of Akt phosphorylation, which was abrogated by bortezomib in a dose-dependent manner. These *in vitro* results were also confirmed *in vivo*, as bortezomib significantly suppressed rapamycin-induced activation of Akt, which in turn enhanced the rapamycin-induced inhibition of tumor growth and pulmonary pulmonary metastasis in orthotopic HCC mice. These data suggested that it is rational to combine rapamycin with bortezomib for targeted HCC therapy.

Although rapamycin has been previously reported to induce cell apoptosis [35], rapamycin treatment alone did not induce apoptosis of HCCLM3 and SMMC7721 cells in this study. However, we demonstrated that the combination of rapamycin and bortezomib produced greater apoptotic activity in HCCLM3 and SMMC7721 cells than either agent alone. This synergistic apoptotic effect was at least partially due to the suppression of rapamycin-induced Akt activation by bortezomib, which may overcome rapamycin resistance. In addition, our data demonstrated that the combination of rapamycin and bortezomib significantly enhanced the expression of p53 mRNA, which may lead to an irreversible apoptotic commitment. The activation of p53 may also contribute to apoptosis by inhibiting Akt phosphorylation [36,37]. Once activated, p53 induces up-regulation of p21 and Bax, which mediate different aspects of p53 function on proliferation arrest and apoptosis [38,39]. Our data suggested that the pro-apoptotic effect of rapamycin plus bortezomib involves elevated transcriptional activity of p53. P53 inhibitor PFT-α can attenuate the pro-apoptotic effect of the combined treatment. Based on our results, we propose that the combined treatment induced apoptosis in HCCLM3 cells is mainly, if not completely, dependent on p53 activity.

The expression of p27 mRNA in HCCLM3 cells was also significantly up-regulated by rapamycin or the combination of rapamycin and bortezomib. Moss *et al.* reported that rapamycin increased p27 levels, which inhibited migration of human umbilical vein endothelial cells (HUVECs) and human coronary artery endothelial cells (HCAEC). Silencing of p27 with small interfering RNA blocked the effects of rapamycin on migration and tube formation [40]. Although the link between mTOR inhibition and cell migration need further investigation, the increased level of p27 induced by rapamycin or the combination treatment may be a potential molecular mechanism involved in the regulation of HCCLM3 cell migration.

Furthermore, we examined the effects of rapamycin and/or bortezomib in orthotopic tumor model of HCC. Rapamycin alone significantly inhibited HCCLM3-R tumor growth. Bortezomib produced less growth inhibition in HCCLM3-R tumors as compared to rapamycin, even though bortezomib induced higher level of apoptosis in HCCLM3 cells *in vitro*. The combination of rapamycin and bortezomib generated greater tumor growth inhibition than either agent alone, with the rate of tumor growth inhibition index as high as 72.4%. Moreover, only 16.6% of mice (1/6) developed pulmonary metastasis in the combination treatment group. Our data demonstrated that bortezomib down-regulated the rapamycin-induced activation of Akt in tumor tissues, which may be one of mechanisms of the novel synergistic antitumor effects. The average proliferation index was dramatically decreased from 62.8 ± 12.7% in rapamycin treatment group to 29.8 ± 6.5% in the group that received both rapamycin and bortezomib. Rapamycin has showed antiangiogenic activities *in vivo* not only by decreasing the production of VEGF but also by inhibiting the response of vascular endothelial cells on stimulation by VEGF *via* inhibiting of mTOR [41]. One mechanism of bortezomib-induced tumor growth inhibition identified in previous studies is also angiogenesis inhibition [42]. In our study, rapamycin, but not bortezomib, significantly reduced microvessel density in orthotopic HCC tumors. The combination of rapamycin and bortezomib amplified the rapamycin-induced inhibition of tumor angiogenesis from 33.6% to 53.0%.The mechanism of synergistic inhibition of angiogenesis by these agents is still unclear and needs further investigation.

In this study, we demonstrated the synergistic effects of rapamycin and bortezomib in inhibiting cell growth, inducing cell apoptosis, and suppressing cell migration and invasion *in vitro*. Rapamycin alone or in combination with bortezomib, strongly inhibited HCC growth and pulmonary metastasis *in vivo*. Bortezomib overcame rapamycin resistance in HCC cells partly through inhibition of the PI3K/Akt pathway activated by rapamycin. In summary, this study provides preclinical evidence that the combination of rapamycin and bortezomib could be a novel and promising therapeutic approach to the treatment of HCC, which warrants further investigation in a clinical setting.

## Conclusions

Rapamycin combined with bortezomib exhibited synergistic antitumor effects on experimental HCC growth and metastasis. It should be a novel and promising therapeutic approach to the treatment of HCC, and worth further studies in patients with HCC, especially disease at advanced stages.

## Competing interests

The authors declare that they have no competing interests.

## Authors’ contributions

CW, DMG, KG and YKL organized the study. CW and KJ carried out the cell culture and molecular studies, and participated in the data analysis. CW, DMG and WZW contributed to the establishment of orthotopic tumor model. LS, HS and HYS participated in the Western blot analysis. GZJ, XNK and YL participated in the design and coordination of the study. CW, KG, CS and YKL contributed to the interpretation of the results and helped write the manuscript. All authors read and approved the final manuscript.

## Pre-publication history

The pre-publication history for this paper can be accessed here:

http://www.biomedcentral.com/1471-2407/12/166/prepub

## Supplementary Material

Additional file 1 ** Table S1.** RT-PCR primer sequences and reaction conditions. Click here for file

Additional file 2 ** Figure S1.** Combined treatment with rapamycin and bortezomib inhibits SMMC7721 proliferation. (A) Proliferation of SMMC7721 cells was evaluated by using Cell Counting Kit-8 (CCK-8) at indicated time points. (B) SMMC7721 cells were treated with rapamycin (10 ng/ml), bortezomib (100 nM) or both agents. Cell cycle analysis was carried out after 24 h. One representative experiment of three is shown. Click here for file

Additional file 3 ** Figure S2.** Rapamycin plus bortezomib causes enhanced apoptosis. (A) SMMC7721 cells were treated with rapamycin (10 ng/ml), bortezomib (100 nM) or the combination for 24 or 48 h and stained with Hoechst 33342 (magnification, ×400). (B, C) The quantification of apoptotic cells induced by rapamycin and bortezomib was further confirmed by flow cytometry analysis. ^*^P < 0.01, *versus* control group; ^**^P < 0.001, *versus* control group; ^#^*P* < 0.05, *versus* bortezomib treatment group at 48 h. Click here for file

Additional file 4 ** Figure S3.** Bortezomib significantly suppressed rapamycin mediated Akt phosphorylation in SMMC7721 cells. (A, B) SMMC7721 cells were cultured in control media, rapamycin (10 ng/ml), bortezomib (100 nmol/L), or rapamycin and bortezomib for 24 h. ^*^P < 0.05, *versus* control group; ^**^P < 0.01, *versus* control group; ^#^*P* < 0.01, *versus* rapamycin treatment group. Click here for file

Additional file 5 ** Table S2.** Lung metastasis in HCCLM3-R xenograft model. Click here for file
