# Supplementary material for: Novel synergistic antitumor effects of rapamycin with bortezomib on hepatocellular carcinoma cells and orthotopic tumor model
Source: BMC Cancer. 2012 May 4;12:166. doi: 10.1186/1471-2407-12-166 (PMC3469344; doi:10.1186/1471-2407-12-166)
Supplement: Additional file 1 — Table S1. RT-PCR primer sequences and reaction conditions. [file 1471-2407-12-166-S1.doc]

**Supplementary Table 1 RT-PCR primer sequences and reaction conditions**

| Gene | Primer sequences (5’ → 3’) | Annealing (ºC) | Cycles | Size (bp) |
| --- | --- | --- | --- | --- |
| p53 | AGCCACATTCTAGGTAGGG | 51 | 35 | 106 |
|  | TGCCAGCATTTCACAGAT |  |  |  |
| p27 | TACGAGTGGCAAGAGGTG | 56 | 35 | 192 |
|  | CGGATCAGTCTTTGGGTC |  |  |  |
| p21 | AGCAGCGGAACAAGGAGT | 52 | 35 | 112 |
|  | TTAGTGCCAGGAAAGACAAC |  |  |  |
| Bim | TTACCAAGCAGCCGAAGA | 52.3 | 35 | 173 |
|  | TAATGGCACCAGGAGACC |  |  |  |
| Bid | GCCGTCCTTGCTCCGTGAT | 54 | 35 | 186 |
|  | ATGCCAGGGCTCCGTCTA |  |  |  |
| Bak | GAGCAGGTAGCCCAGGACA | 56.1 | 35 | 194 |
|  | TAGCGTCGGTTGATGTCG |  |  |  |
| Bcl-2 | TCCAATCCTGTGCTGCTA | 50 | 35 | 172 |
|  | ACTCTGTGAATCCCGTTT |  |  |  |
| Bcl-xl | GGCAGGCGACGAGTTTGA | 54.2 | 35 | 197 |
|  | CCTTGTCTACGCTTTCCAC |  |  |  |
| Bax | TTTTGCTTCAGGGTTTCAT | 54.5 | 35 | 114 |
|  | ACACTCGCTCAGCTTCTTG |  |  |  |
| GAPDH | GACCTGACCTGCCGTCTA | 53.5 | 35 | 148 |
|  | AGGAGTGGGTGTCGCTGT |  |  |  |
