# Supplementary figures and images for: Novel synergistic antitumor effects of rapamycin with bortezomib on hepatocellular carcinoma cells and orthotopic tumor model
Source: BMC Cancer. 2012 May 4;12:166. doi: 10.1186/1471-2407-12-166 (PMC3469344; doi:10.1186/1471-2407-12-166)

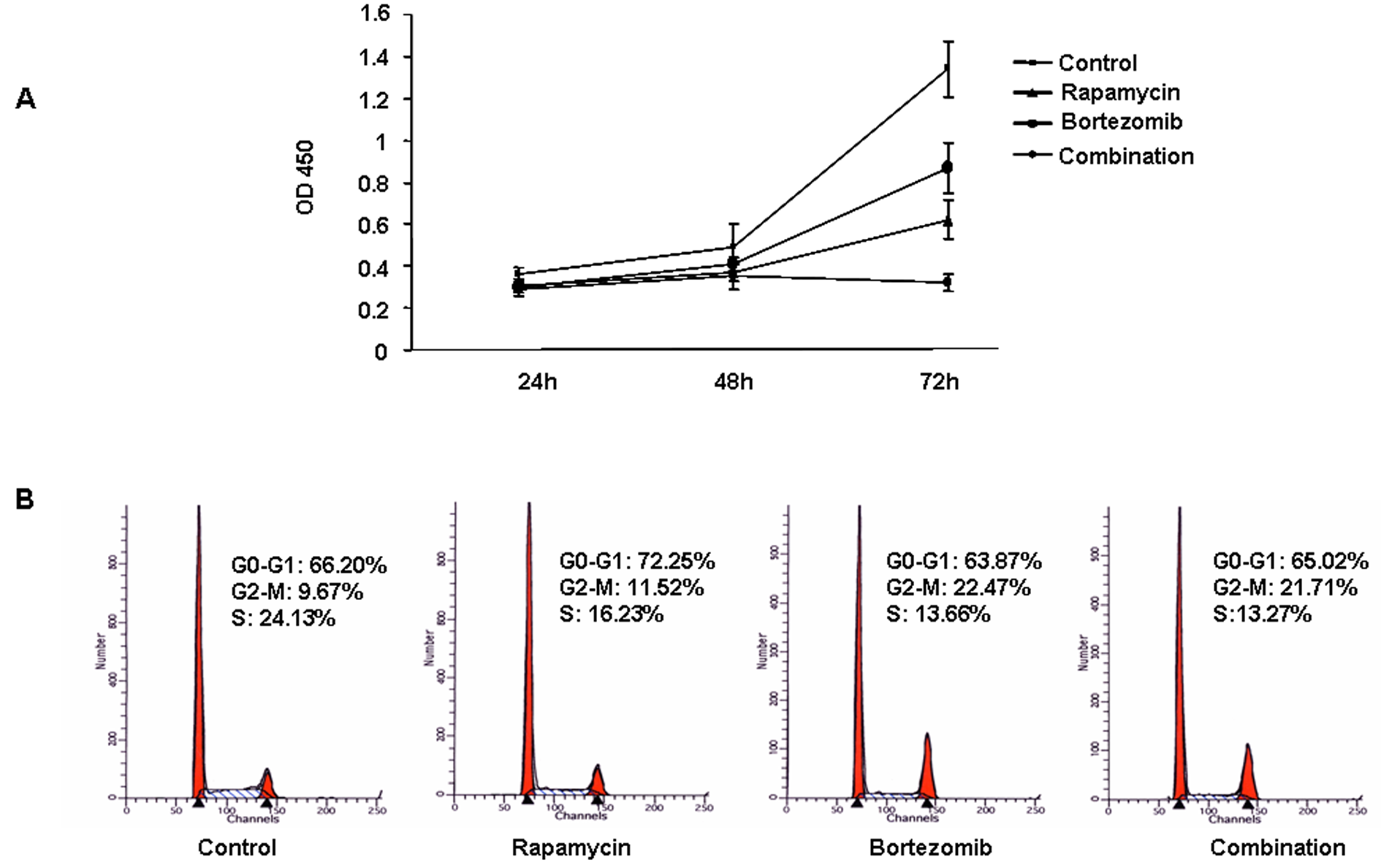

Supplement: Additional file 2 — Figure S1. Combined treatment with rapamycin and bortezomib inhibits SMMC7721 proliferation. (A) Proliferation of SMMC7721 cells was evaluated by using Cell Counting Kit-8 (CCK-8) at indicated time points. (B) SMMC7721 cells were treated with rapamycin (10 ng/ml), bortezomib (100 nM) or both agents. Cell cycle analysis was carried out after 24 h. One representative experiment of three is shown. [file 1471-2407-12-166-S2.tiff]

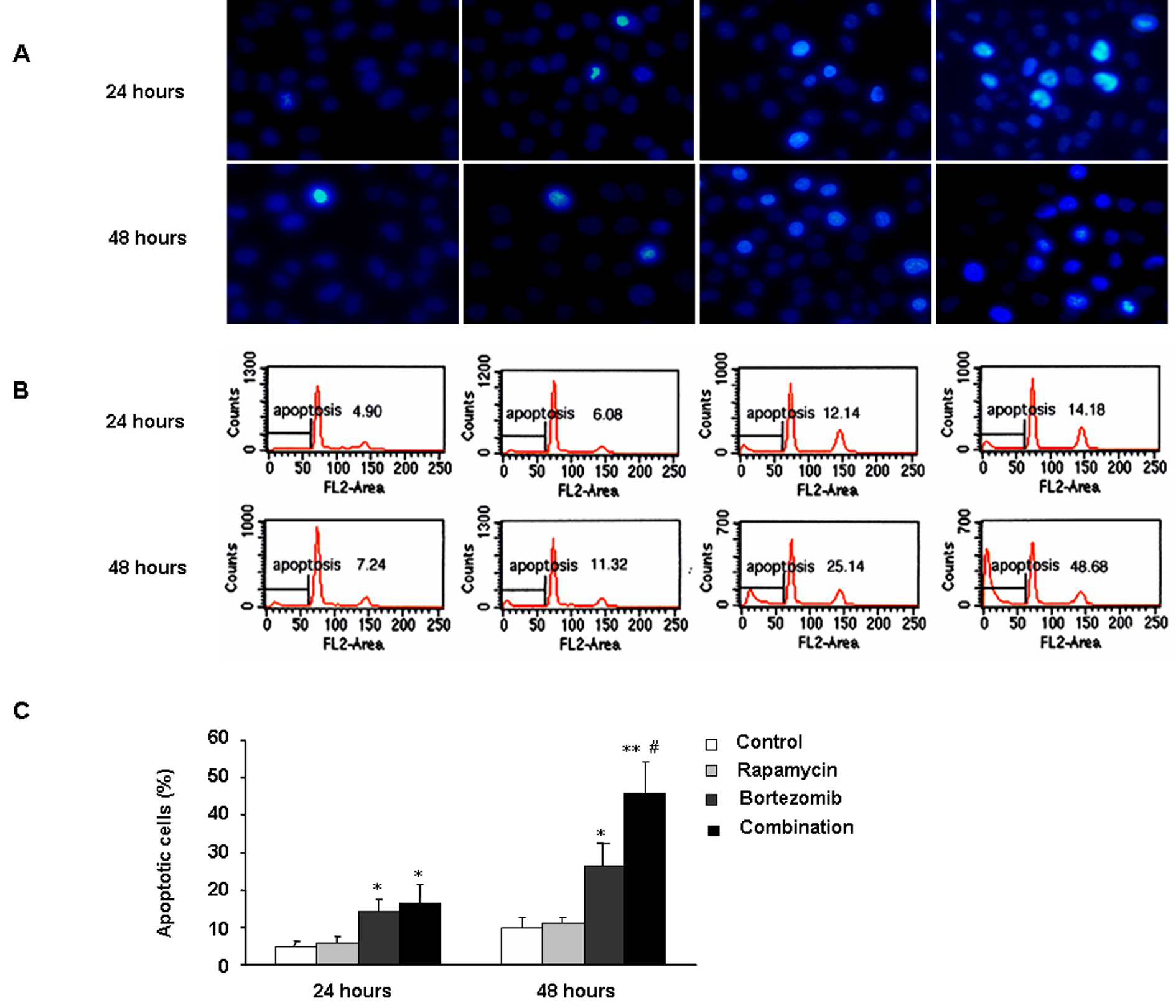

Supplement: Additional file 3 — Figure S2. Rapamycin plus bortezomib causes enhanced apoptosis. (A) SMMC7721 cells were treated with rapamycin (10 ng/ml), bortezomib (100 nM) or the combination for 24 or 48 h and stained with Hoechst 33342 (magnification, ×400). (B, C) The quantification of apoptotic cells induced by rapamycin and bortezomib was further confirmed by flow cytometry analysis. *P < 0.01, versus control group; **P < 0.001, versus control group; #P < 0.05, versus bortezomib treatment group at 48 h. [file 1471-2407-12-166-S3.tiff]

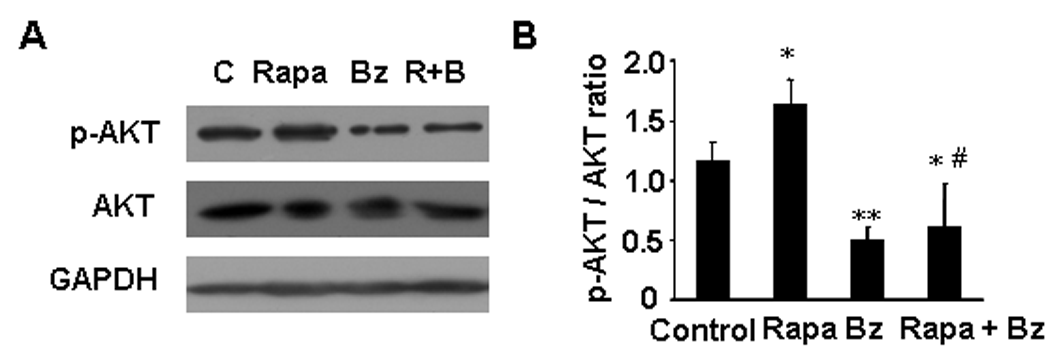

Supplement: Additional file 4 — Figure S3. Bortezomib significantly suppressed rapamycin mediated Akt phosphorylation in SMMC7721 cells. (A, B) SMMC7721 cells were cultured in control media, rapamycin (10 ng/ml), bortezomib (100 nmol/L), or rapamycin and bortezomib for 24 h. *P < 0.05, versus control group; **P < 0.01, versus control group; #P < 0.01, versus rapamycin treatment group. [file 1471-2407-12-166-S4.tiff]
