# Supplementary material for: Novel synergistic antitumor effects of rapamycin with bortezomib on hepatocellular carcinoma cells and orthotopic tumor model
Source: BMC Cancer. 2012 May 4;12:166. doi: 10.1186/1471-2407-12-166 (PMC3469344; doi:10.1186/1471-2407-12-166)
Supplement: Additional file 5 — Table S2. Lung metastasis in HCCLM3-R xenograft model. [file 1471-2407-12-166-S5.doc]

**Supplementary Table 2 lung metastasis in HCCLM3-R xenograft model**

|  | Control | Rapamycin | Bortezomib | Combination |
| --- | --- | --- | --- | --- |
| Mice-bearing pulmonary metastasis / total mice | 100% | 50.0% | 66.6% | 16.6% * |
| Median No. of pulmonary nodules | 55 | 17.5 | 30 | 0 |

*P* < 0.05, Fisher’s Exact test versus control group.
